# Sex Differences in Problem Alcohol Use in High School as a Function of Recent Sexual Violence Victimization or Perpetration

**DOI:** 10.1007/s10896-019-00116-5

**Published:** 2019-12-10

**Authors:** Christal L. Badour, Samuel C. Bell, Emily R. Clear, Heather M. Bush, Ann L. Coker

**Affiliations:** 1 Department of Psychology, University of Kentucky, College of Arts & Sciences, Lexington, KY 40506 USA; 2 Office of Research Integrity, University of Kentucky, Lexington, KY 40536 USA; 3 College of Public Health, University of Kentucky, Lexington, KY, 40536 USA; 4 College of Public Health, University of Kentucky, Lexington, KY, 40536 USA; 5 Dept of Obstetrics & Gynecology, College of Medicine,, University of Kentucky, Lexington, KY 40536 USA

**Keywords:** Sexual violence perpetration, Problem alcohol use, Adolescents, Sex differences

## Abstract

To investigate sex differences in associations between sexual violence victimization (SVV), sexual violence perpetration (SVP), and binge drinking and/or alcohol problems among high school students. While SVV has been linked to problem alcohol use among young women, little research has addressed the unique associations of SVV and SVP on alcohol use/problems within both sexes. A cross-sectional analysis of 16,992 high school students’ self-reports of past-year SVP and SVV was used where SVV/SVP was defined by three tactics (sexual coercion, drug/alcohol-facilitated or incapacitated sex, and physically forced sex). Alcohol measures included past-month binge drinking and past-year alcohol problems. Rates of SVV were twice as high in females (21.2% vs. 13.3%), and SVP rates were twice as high in males (10.9% vs. 5.2%). SVV and SVP were each associated with an increased rate of current binge drinking and problem alcohol use for both sexes, across increasing numbers of SV tactics and within each of three tactics. After controlling for demographic and other risk factors including SVP, drug/alcohol-facilitated or incapacitated SVV was more strongly linked to binge drinking and alcohol problems among females. SVP was more strongly linked to binge drinking and alcohol problems among males (adjusting for SVV and other covariates). No sex differences emerged in associations between coerced or physically forced SVV/SVP and alcohol-related outcomes. Both SVV and SVP are associated with an increased likelihood of binge drinking and alcohol problems for males and females. Important sex differences emerged when SV tactics are considered.

## Introduction

The Centers for Disease Control and Prevention (CDC) defines sexual violence (SV) as “a sexual act that is committed or attempted by another person without freely given consent of the victim or against someone who is unable to consent or refuse” (Basile et al. 2014). Tactics included within the definition are 1) sexual coercion (i.e., pressure for sex in a non-physical way), 2) physically forced sex, and 3) alcohol/drug-facilitated (i.e., involving perpetrator-administered drugs, alcohol, or other intoxicants that result in loss of consciousness or impaired ability to control behavior) or incapacitated (i.e., involving impairment that occurs when the victim has voluntarily used alcohol or other drugs) sex (Black et al. 2011). Lifetime and current rates of sexual violence victimization (SVV) are higher among women than men (Black et al. 2011). Sexual violence perpetration (SVP) has more recently been enumerated in population-based samples where rates are two to three times higher among men than women (Krahé et al. 2015). Similar sex differences in SVP were found among children (Hamby et al. 2013) and adolescents (Ybarra and Mitchell 2013). Alcohol use frequently co-occurs with SVV and SVP for both women and men. Indeed, approximately half of all SV incidents involve alcohol consumption by the victim, perpetrator, or both (Abbey 2011; Abbey et al. 2004b; Testa 2002).

## Sexual Violence Victimization (SVV) and Alcohol Use

History of SVV has been consistently linked to increased binge drinking (Behnken et al. 2010; Howard and Wang 2005; Loh et al. 2014; McCauley and Calhoun 2008); with the majority of this evidence focusing on adolescent or young adult females. For example, relative to non-victims, females experiencing SVV in childhood have been found to have an increased risk of binge drinking (Jasinski et al. 2000), and excessive drinking through middle adulthood via increased likelihood of an alcohol use disorder in early adulthood (Widom et al. 2007). Among adult females from three different age groups, Walsh et al. (2014) observed that lifetime SVV was associated with an increased risk for past-year binge drinking. Rates of binge drinking also increased with a new SVV incident (Walsh et al. 2012). Prospective research is mixed regarding the temporal nature of the association between SVV and alcohol use, with some findings suggesting SVV increases risk for or exacerbates problem drinking (Norris et al. 2019; Parks et al. 2014), others finding that binge/heavy drinking and alcohol use disorders increase risk for SVV (Gidycz et al. 2007; Mouilso et al. 2012), and others still suggesting this relation is bi-directional (Bryan et al. 2016; Kaysen et al. 2006).

Considerably less is known of the association between SVV and alcohol use among males, as the majority of studies on SVV include only female participants or few males (Budd et al. 2019). Recent nationally-representative data suggests that although among youth, females aged 14–17 are at the highest risk for experiencing SVV (past-year SVV = 16.4%), males in this age group also report high rates of past-year SVV 9.4% (Finkelhor et al. 2015). Among adult males, history of SVV in childhood has been linked to younger age of first intoxication, which in turn, has been shown to predict higher alcohol consumption in adulthood (Schraufnagel et al. 2010). Several studies have documented similarly increased risk for mental health and substance use problems among males and females exposed to SVV in childhood including increased risk of binge drinking (Howard and Wang 2005), alcohol problems, (Dube et al. 2005) and alcohol dependence (Fergusson et al. 2013). Others have found that adolescent boys with a history of SVV experience disproportionately increased risk for problems related to alcohol and drug use compared to girls experiencing SVV (Garnefski and Arends 1998). In contrast, Loh et al. (2014) found in a sample of 44,610 9th grade students in Germany that lifetime SVV was only correlated with binge drinking among females, while lifetime physical victimization was associated with binge drinking among males.

## Sexual Violence Perpetration (SVP) and Alcohol Use

Important research provides evidence that measures of distal (i.e., general alcohol consumption over the past year/month, alcohol dependence), proximal (i.e., frequency/quantity used in potential sexual situations), and event-level alcohol use (i.e., drinking during an SV incident) have all been linked to an increased risk of SVP (Abbey 2011; Abbey et al. 2014; Crane et al. 2016), particularly for adult men (Tharp et al. 2013). The quantity of alcohol consumed on drinking days during high school has also been shown to prospectively predict single (but not multiple) incidents of SVP during college for young adult males (Wilhite and Fromme 2017). Moreover, male college freshman who engage in heavy drinking are more likely to report a history of SVP prior to college compared to males who do not report heavy drinking (Salazar et al. 2018). Extant research on SVP and alcohol use is primarily limited to males, despite the fact that females also engage in SVP (Schatzel-Murphy et al. 2009). Moreover, SVV and SVP are typically examined separately. This failure to examine a third category of individuals who have both experienced SVV *and* engaged in SVP may have inadvertently resulted in important gaps in our understanding of both the antecedents and consequences of SV (Richards et al. 2017).

## Current Study

The aim of the present study was to examine current binge drinking (past-month) and alcohol problems (past 12-months) in a large population-based sample of male and female high school students as a function of past 12-month SVV and SVP, number of SV tactics used or experienced, and type of SV tactic (i.e., coerced, physically forced or alcohol/drug facilitated or incapacitated sex). There has been a dearth of research addressing SVV among males, SVP among females, and the combined effects of both SVV and SVP by sex. Moreover, with few exceptions (e.g., Fernández-Fuertes et al. 2018; Ybarra and Mitchell 2013), very little research has examined the frequency or impact of SV by tactic among male and female youth. Finally, this research was conducted in an adolescent sample which extends the majority of research on alcohol use and SV which has been conducted among college students (Abbey 2011).

We hypothesized that, for both male and female adolescents, those experiencing (SVV) or using (SVP) violence would report more frequent binge drinking and alcohol problems compared to adolescents reporting no SV (Hypothesis 1). Further, binge drinking and alcohol problems would be more strongly associated with SVP among males compared to females (Hypothesis 2). The rationale for this hypothesis comes from theoretical work aimed at understanding alcohol’s role in SVP, which has been almost exclusively focused on male perpetrators. As noted by Abbey (2011) SV perpetrators are a heterogeneous group with differing risk factors; however, alcohol use frequently co-occurs with SV (Tharp et al. 2013) and specifically with SVP among males (Testa 2002). In particular, excessive alcohol use may impair executive functioning, and response inhibition, such that cues that typically inhibit sexually aggressive behavior (e.g., morality, empathy, concern for future consequences, signs that partner does not want to have sex) may become less salient compared to immediate feelings of sexual arousal, frustration or entitlement while intoxicated (Abbey 2011; Gallagher et al. 2010). Additional support for this hypothesis comes from findings that males who engage in SVP when drinking tend to drink more (Abbey 2011). Fed in part by culture norms of victim blaming, some male perpetrators perceive female alcohol use as an indicator of interest in sexual activity (Abbey et al. 2000) or that female alcohol use makes someone a convenient target of assault (Lisak and Miller 2002).

Finally, drug/alcohol-facilitated or incapacitated SV was hypothesized to be the tactic most strongly associated with binge drinking and alcohol problems for both sexes and for both SVP and SVV (Hypothesis 3). This hypothesis was based primarily on evidence that drug/alcohol-facilitated or incapacitated SVV among college or community-residing women appears to demonstrate a uniquely strong relationship with binge and heavy drinking behavior compared to other forms of SVV (Abbey et al. 2004a, b; McCauley et al. 2009; Testa et al. 2003). However, at least one study found that incapacitated SVV was both concurrently and prospectively linked to higher alcohol consumption and alcohol-related problems among both male and female college students (Kaysen et al. 2006). It has been suggested that a lifestyle of heavier drinking may increase risk, in particular, for drug/alcohol-facilitated or incapacitated SVV (McCauley et al. 2009), and experiencing this form of SVV may be uniquely linked to subsequent increases in alcohol use behavior and associated consequences (Kaysen et al. 2006). Although this work has focused on SVV, it stands to reason that a similar relationship may emerge for SVP. Exploring the SV tactics used in combination with patterns of alcohol use by both victims and perpetrators may help to better understand whether and how perpetrators may target alcohol- or drug- impaired victims.

## Method

This cross-sectional analysis was based on data collected in 2011 as part of a five-year cluster randomized controlled trial to evaluate the effectiveness of a bystander intervention where anonymous surveys were conducted annually from 2010 to 2014 (Coker et al. 2017). Analyses were only conducted for one year to eliminate student duplications with anonymous surveys over 5 years. Data from 2011 was selected because this was the second year of data collection, providing a baseline for the majority of 10th–12th grade high school students surveyed. This also limited telescoping of violence reports by providing bounding with the prior year’s data collection (Tourangeau and McNeeley 2003). Briefly, telescoping of annual violent events can occur if no baseline measure is obtained because participants may include experiences in past years with reports queried for the past 12 months. This year was also selected because the intervention for the parent trial had not been fully implemented in 2011. Statistical analyses adjusted for the randomized controlled trial design with randomization by school.

## Participants and Procedure

As described elsewhere (Coker et al. 2017), high school students attending one of 26 high schools were invited to complete a 99-item self-administered survey. Study personnel administered surveys in either a classroom administration setting during a selected class or in a school group administration during one class period when all students were surveyed. Elements of the assent form were read to all students by study staff. Students were encouraged to read the assent form in the survey booklet and decide for themselves whether they wished to participate. All surveys in this study were administered between February and May 2011. Resources including websites and toll-free numbers for national agencies to address domestic violence, sexual assault, depression or suicide ideation, were included at the end of the survey booklet should students find this information helpful. National hotline information was also provided on pencils the students used to complete the survey. The study protocol was approved by the University of Kentucky Institutional Review Board 09–0680-F1V.

### Parental Passive Consent

Study staff mailed a letter describing the study purpose and information to the parents or guardians of all enrolled students at least two weeks prior to surveying. If parents/guardians did not want their student to participate they were instructed to call or email study staff who worked with school administrators to ensure that these students were not surveyed.

### Student Response Rates

Of the 21,608 students present in the 26 schools and completing a scantron form, 2625 refused (12.15%; 11.65% student refusals, 0.5% parental refusals). Another 1857 students were excluded due to incomplete responses defined as completing less than 30 of the 99 items (partial refusals). Of the remaining 17,126 students, 38 were excluded because they were not in grades 9–12 or did not disclose their sex (male or female). The remaining students were missing data on either binge drinking or alcohol problems (*n* = 44) or current relationship status (*n* = 35). The final analytic sample included 16,992 students. There were no significant demographic differences between those who agreed and completed the survey and those who did not with the following exception: males were twice as likely (*p* < .0001) as females to refuse to complete the survey.

The following are demographic descriptors of the final analytic sample: 45% were male, 82% were of non-Hispanic white race, 43% received a free or reduced price school meal, 28% were freshmen, 28% sophomore, 25% juniors, and 18% were seniors, 86% were exclusively attracted to the opposite sex, 79% were in a dating relationship, and 50% were sexually active.

### Sexual Violence Victimization and Perpetration in the Past 12 Months

SV questions were prefaced with the following introduction: “The next questions are about sexual activities. Some of the questions might make you uncomfortable. Remember that this survey is anonymous. Your name will not be linked to your answers. You may also skip questions that make you uncomfortable.” Three items were used to measure SVV and were based on the CDC SV definition (Black et al. 2011) using the following stem: “In the last 12 months, how many times have YOU…: 1) Had sexual activities even though you didn’t really want to because either they threatened to end your friendship or romantic relationship if you didn’t or you felt pressured by the other person’s constant arguments or begging (sexual coercion); 2) Had sexual activities when you didn’t want to because the other person threatened to use or used physical force (like twisting your arm, holding you down) if you did not agree (physically forced sex); and 3) Had sexual activities when you didn’t want to because you were drunk or on drugs (alcohol/drug facilitated or incapacitated sex).” Response options for all items ranged from never, 1–2 times, 3–5 times, 6–9 times and 10 or more times, and yes, but not in the past 12 months. Indicator variables were created for each item to represent the proportion reporting each tactic occurring one or more times in the past 12 months (all dichotomous yes/no variables).

The same three items were rephrased to determine SVP: “In the last 12 months, how many times have YOU… 1) Had sexual activities with a high school student because you either threatened to end your friendship or romantic relationship if they didn’t or because you pressured the other person by arguing or begging? (sexual coercion), 2) Had sexual activities with another high school student by threatening to use or used physical force (twisting their arm, holding them down, etc.)?, 3) Had sexual activities with another high school student because she/he was drunk or on drugs (alcohol/drug facilitated or incapacitated sex).” The ordinal response options for these items were the same as those for SVV. Indicator variables were again created for those who reported they had committed each tactic one or more times. SVV questions were asked before SVP questions.

In addition to variables indicating the frequency of any SVV and SVP and by form (e.g., coerced sex, physically forced sex, and alcohol/drug facilitated or incapacitated sex), another created variable measured the number of SV forms either used (SVP) or experienced (SVV); this variable ranged from 0 to 3. Lastly, to distinguish SVV from SVP a variable was created to address combinations of SV as follows: 1) both SVV and SVP, 2) SVP alone (no SVV), 3) SVV alone (no SVP) and 4) the comparison group of no SVP nor SVV.

### Current Binge Drinking and Alcohol Problems

The following question was used to define self-reported binge drinking in the past month: “In the past month, on how many days did you have 4 or 5 or more drinks of alcohol in a row within a couple of hours.” Response options were “I never drink (=0), 0 days (=1), 1-2 days (=2), 3-9 days (=3), 10-19 (=4), 20-31 days (=5)”. A dichotomous variable was created to define any binge drinking (1–31 days binge drinking in the prior month); 25.1% of all participants disclosed binge drinking in this time frame).

Alcohol problems were defined using responses to the following four questions based on items from the Rutgers Alcohol Problem Index (White and Labouvie 1989): “In the past 12 months, have you: 1) gotten into a fight or done poorly at work or school due to drinking alcohol; 2) missed a day or more of work or school due to drinking alcohol; 3) afterwards, been unable to remember things that happened while you were drinking (things you would normally remember); and 4) done things when drinking that you normally would not do and you now regret doing?” A dichotomous variable was initially created to define any alcohol problem in the past 12 months; 28.7% scored as having one or more alcohol problems).

Because any binge drinking in the past month was highly correlated with having one or more alcohol problems in the past 12 month (Cochran-Mantel-Haenszel Chi square = 4813.79, *p* < .0001), we opted to refine our definition of alcohol problems from one or more to two or more alcohol problems to better differentiate recent binge drinking from alcohol problems over the past 12 months.

### Demographics and Other Risk Factors

Several demographic variables were included in the survey. Sex (male or female) and grade in school (9–12) were analyzed as asked. Given the limited racial and ethnic diversity of Kentucky, a simplified dichotomous indicator of white compared to all other racial/ethnic categories was used. Gender identity was not assessed but sexual attraction was queried. Sexual attraction was assessed with the following item: “People are different in their sexual attraction to other people. Which best describes your feelings?” The response options were: 1) only attracted to females, 2) mostly attracted to females, 3) equally attracted to females and males, 4) mostly attracted to males, 5) only attracted to males, and 6) not sure. Students who reported that they were male and were only attracted to females and those that reported that they were female and were only attracted to males were coded as being exclusively attracted to the other sex. All others, were coded as not exclusively attracted to the opposite sex. Current dating relationship status was addressed with the following question: “During the past 12 months, have you been in a relationship with a boyfriend or girlfriend? By a relationship we mean either having a partner for planned event like a school dance or going to the movies, having a sex partner, or hanging out in a group with a boyfriend or girlfriend.” Response options were collapsed to indicate whether the student had or had not been in a relationship in the past 12 months. The construct of having witnessed parental partner violence was measured with the item: “In your family, how often did you see or hear one of your parents or guardians being hit, slapped, punched, shoved, kicked or otherwise physically hurt by their spouse or partner?”

## Statistical Analysis

Given the large sample size, alpha was set at *p* < .001 to determine significance for all analyses. Sex (male, female) was investigated as a modifier for the association between SV and the two alcohol-related outcomes. Tests of effect modification of the association between SVV and SVP and binge drinking by sex were first conducted using the Breslow-Day test for homogeneity of odds ratios Chi-square tests to identify modification by sex. Sex differences in rates of any form of SVV and SVP overall and by SV form tactic were estimated using a series of Chi-square tests (see Table 1). To identify confounders, demographic or violence risk factors were correlated with SVV and SVP by sex again using a Chi-square test. Primary analyses estimated the association between SVP and the two alcohol-related measures and adjusting for confounders.

**Table 1:** Frequency of sexual violence victimization (SVV) and perpetration (SVP) by sex

Time Frame = past 12 months	Males *N* = 7599# (%)	Females *N* = 9393# (%)	Sex difference in SV rates: χ^2^_df_^*p* value^
Any Sexual Violence			
SV Perpetration	823 (10.8)	489 (5.2)	186.49 _1_^*p* < .0001^
NO SVP	6776 (89.2)	8904 (94.8)	REF
SV Victimization	1007 (13.3)	1993 (21.2)	183.35 _1_^p < .0001^
NO SVV	6592 (86.7)	7400 (78.8)	REF
Number of SVP forms			214.15 _3_^p < .0001^
3	187 (2.4)	71 (0.8)	
2	179 (2.4)	73 (0.8)	
1	457 (6.0)	345 (3.6)	
0	6776 (89.2)	8904 (94.8)	
Number of SVV forms			229.37 _3_^p < .0001^
3	176 (2.3)	173 (1.8)	
2	257 (3.4)	534 (5.7)	
1	574 (7.6)	1286 (13.7)	
0	6592 (86.7)	7400 (78.8)	
*ANY Sexual Violence*			616.92 _3_^<.0001^
SVV & SVP	530 (7.0)	358 (3.8)	
SVV only	477 (6.3)	1635 (17.4)	
SVP only	293 (3.9)	131 (1.4)	
NO SVV nor SVP	6299 (82.9)	7269 (77.4)	
*By Tactic*			
Sexual Coercion			
Coerced SVP	400 (5.3)	188 (2.0)	133.82 _1_^p < .0001^
No Coerced SVP	7199 (94.7)	9205 (98.0)	REF
Coerced SVV	638 (8.4)	1482 (15.8)	209.61 _1_^p < .0001^
No Coerced SVV	6961 (91.6)	7911 (84.2)	REF
Physically Forced Sex			
Forced SVP	327 (4.3)	130 (1.4)	136.77 _1_^p < .0001^
No Forced SVP	7272 (95.7)	9263 (98.6)	REF
Forced SVV	355 (4.7)	517 (5.5)	5.98 _1_^*p* = .01^
No Forced SVV	7244 (95.3)	8876 (94.5)	REF
Drug/Alcohol-facilitated or Incapacitated Sex			
SVP	649 (8.5)	386 (4.1)	144.19 _1_^p < .0001^
No SVP	6950 (91.5)	9007 (95.9)	REF
SVV	623 (8.2)	874 (9.3)	6.40 _1_^p = .01^
No SVV	6976 (91.8)	8519 (90.7)	REF

*p*-values < .001 were determined to be statistically significant given the large sample size

Status of SVP (and SVV) was used as the primary exposure (independent variable) with demographic confounders in two models where binge drinking and alcohol problems were included as dependent variables (Tables 2 and 3, respectively). Comparisons were made using generalized estimating equations, which allow for the relaxation of independence conditions (students attending the same school). These models included adjustment for identified confounders and the parent study design by incorporating the condition as a covariance and including the sampled high school (*n* = 26) in multivariable logistic regression (PROC Genmod, link = log dist = bin type3; repeated subject = code/type = exch). All analyses were conducted using Statistical Analysis Software, SAS, version 9.4; SAS Institute; Cary, North Carolina.

**Table 2:** Sexual Violence Victimization (SVV), Perpetration (SVP), and Past 30-Day Binge Drinking by Sex

Time Frame = past 12 months	Males *N* = 7599		Females *N* = 9393		Test of Sex * SV Interaction χ^2 (p value)^
	% Binge Drinking, Mean (*SE*)	aPRR (95% CI)	% Binge Drinking, Mean (*SE*)	aPRR (95% CI)	
Any Sexual Violence	*n* = 2004 (26.4%)		*n* = 2266 (24.1%)		
Any SVP *	39.17 (.016)	1.84 (1.65–2.05)	32.09 (.019)	1.56 (1.42–1.72)	3.01 ^*p* = .08^
NO SVP	21.33 (.010)	1.00 REF	20.54 (.007)	1.00 REF	
Any SVV **	34.23 (.013)	1.40 (1.26–1.56)	35.91 (.013)	1.96 (1.81–2.12)	12.61 ^*p* < .0001^
NO SVV	24.40 (.012)	1.00 REF	18.36 (.010)	1.00 REF	
Number of SVP forms (Adjusting for # SVV forms)					
3	47.97 (.023)	2.12 (1.88–2.41)	33.76 (.034)	1.34 (1.10–1.65)	18.19 ^p < .0001^
2	31.30 (.031)	1.39 (1.15–1.69)	29.60 (.045)	1.18 (0.89–1.56)	
1	44.03 (.020)	1.95 (1.74–2.19)	38.76 (.020)	1.54 (1.38–1.72)	
0	22.54 (.011)	1.00 REF	25.12 (.009)	1.00 REF	
Number of SVV forms (Adjusting for # SVP forms)					
3	40.32 (.022)	1.53 (1.32–1.77)	44.74 (.028)	2.56 (2.24–2.92)	22.12 ^p < .0001^
2	36.29 (.023)	1.38 (1.19–1.59)	37.12 (.022)	2.13 (1.92–2.34)	
1	38.65 (.022)	1.47 (1.31–1.65)	33.54 (.018)	1.92 (1.75–2.11)	
0	26.35 (.015)	1.00 REF	17.47 (.011)	1.00 REF	
Any SV Form Combinations of SVV and SVP					6.16 ^p = NS^
SVV/SVP	43.03 (.015)	2.53 (2.32–2.75)	42.75 (.021)	3.09 (2.77–3.44)	
SVV only	33.52 (.022)	1.97 (1.72–2.25)	29.51 (.009)	2.07 (1.90–2.25)	
SVP only	43.88 (.022)	2.58 (2.33–2.86)	35.79 (.030)	2.51 (2.11–2.99)	
NO SVV or SVP	17.02 (.008)	1.00 REF	14.26 (.007)	1.00 REF	
By SV Tactic					
Sexual Coercion					
SVP *	37.04 .019	1.61 (1.44–1.81)	28.51 (.021)	1.35 (1.19–1.54)	2.91 ^p = NS^
No SVP	22.96 (.009)	1.00 (REF)	21.05 (.007)	1.00 REF	
SVV **	33.33 (.013)	1.31 (1.16–1.48)	30.56 (.015)	1.56 (1.42–1.71)	6.47 ^p = .01^
No SVV	25.52 (.014)	1.00 (REF)	9.64 (.011)	1.00 (REF)	
Physically Forced Sex					
SVP *	37.36 (.018)	1.62 (1.42–1.86)	29.12 (.028)	1.32 (1.11–1.59)	3.23 ^*p* = .07^
No SVP	23.01 (.011)	1.00 (REF)	21.99 (.009)	1.00 (REF)	
SVV **	33.12 (.017)	1.28 (1.10–1.48)	31.09 (.021)	1.51 (1.33–1.71)	5.05 ^*p* = .02^
No SVV	25.96 (.013)	1.00 REF	20.60 (.013)	1.00 REF	
Drug/Alcohol-facilitated or Incapacitated Sex					
SVP *	40.80 (.018)	1.82 (1.62–2.04)	31.93 (.014)	1.29 (1.19–1.39)	17.42 ^p < .0001^
No SVP	22.43 (.011)	1.00 REF	24.82 (.009)	1.00 REF	
SVV **	38.77 (.017)	1.66 (1.51–1.83)	46.67 (.017)	2.81 (2.56–3.09)	14.22 ^*p* = .0002^
No SVV	23.31 (.010)	1.00 REF	16.60 (.008)	1.00 REF	

* Adjusting for Sexual violence victimization (SVV); ** Adjusting for Sexual violence perpetration (SVP)

aPRR = Adjusted prevalence rate ratios adjusting for relationship status (yes v no in a dating or intimate partnered relationship in the past 12 months), race (minority v majority or White), condition (intervention v control randomization), sexual attraction (exclusively attracted to opposite sex vs not exclusively attracted to opposite sex: sexual minority), witnessed parental partner violence (yes v no); 95% CI = (95% confidence interval around the aPRR); REF = referent group; *SE* = standard error. NOTE: aPRR analyses provide comparisons with the referent group being no SVP nor SVV. Test of Interaction Sex x SV measure (Score Statistics For Type 3 GEE Analysis). p-values < .001 were determined to be statistically significant given the large sample size

**Table 3:** Sexual Violence Victimization (SVV), Perpetration (SVP), and Past 12-Month Alcohol Problems by Sex

Time Frame = past 12 months	Males N = 7599		Females N = 9393		Test of Sex * SV Interaction χ^2 (p value)^
	% Alcohol Problems, Mean (*SE*)	aPRR (95% CI)	% Alcohol Problems, Mean (*SE*)	aPRR (95% CI)	
Any Sexual Violence	*n* = 972, 12.8%		*n* = 1193, 12.7%		
Any SVP *	36.33 (.020)	2.63 (2.32–2.98)	27.05 (.011)	1.83 (1.66–2.02)	10.57 ^p = .001^
NO SVP	13.81 (.008)	1.00 REF	14.76 (.006)	1.00 REF	
Any SVV **	36.84 (.019)	2.70 (2.36–3.10)	42.36 (.014)	4.50 (3.97–5.09)	11.91 ^*p* = .0006^
NO SVV	13.62 (.009)	1.00 REF	9.42 (.005)	1.00 REF	
Number of SVP forms (Adjusting for # SVV forms)					26.19 ^p < .0001^
3	38.28 (.035)	2.85 (2.27–3.55)	22.15 (.025)	1.34 (0.99–1.54)	
2	26.12 (.029)	1.94 (1.54–2.44)	19.56 (.022)	1.09 (0.86–1.38)	
1	33.26 (.027)	2.48 (2.172.86)	27.11 (.017)	1.51 (1.34–1.70)	
0	13.44 (.010)	1.00 REF	17.91 (.008)	1.00 REF	
Number of SVV forms (Adjusting for # SVP forms)					38.32 ^p < .0001^
3	36.84 (.031)	2.94 (2.36–3.68)	45.42 (.030)	7.14 (5.92–8.55)	
2	30.62 (.029)	2.44 (1.96–3.03)	33.11 (.025)	5.18 (4.44–6.06)	
1	31.68 (.026)	2.53 (2.21–2.90)	21.89 (.016)	3.42 (3.03–3.89)	
0	12.51 (.010)	1.00 REF	6.40 (.005)	1.00 REF	
Any SV Form Combinations of SVV and SVP					5.59 ^*p* = .03^
SVV/SVP	39.21 (.026)	6.25 (5.37–7.29)	37.23 (.022)	6.96 (6.80–7.96)	
SVV only	23.79 (.016)	3.76 (3.30–4.36)	22.62 (.012)	4.23 (3.77–4.74)	
SVP only	28.19 (.023)	4.50 (3.74–5.41)	21.20 (.034)	3.96 (2.90–5.41)	
NO SVV or SVP	6.27 (.005)	1.00 REF	5.35 (.003)	1.00 REF	
By SVV / SVP Tactic					
Sexual Coercion					
Coerced SVP *	42.21 (.042)	2.43 (1.92–3.06)	29.28 (.024)	1.65 (1.40–1.95)	3.38 ^p = NS^
No Coerced SVP	17.38 (.009)	1.00 (REF)	17.76 (.006)	1.00 REF	
Coerced SVV **	41.56 (.023)	2.35 (1.93–2.87)	39.16 (.018)	2.95 (2.64–3.29)	6.19 ^p = .01^
No Coerced SSV	17.66 (.015)	1.00 (REF)	13.28 (.008)	1.00 (REF)	
Physically Forced Sex					
Forced SVP *	49.63 (.042)	3.08 (2.42–3.91)	31.44 (.029)	1.47 (1.18–1.83)	8.19 ^*p* = .004^
No Forced SVP	16.11 (.011)	1.00 (REF)	21.38 (.011)	1.00 (REF)	
Forced SVV **	37.18 (.030)	1.73 (1.35–2.20)	44.98 (.033)	3.01 (2.58–3.51)	7.46 ^*p* = .006^
No Forced SVV	21.50 (.016)	1.00 REF	14.95 (.008)	1.00 REF	
Drug/Alcohol-facilitated or Incapacitated Sex					
SVP *	40.14 (.021)	2.52 (2.18–2.91)	27.09 (.012)	1.27 (1.18–1.36)	15.22 ^P < .0001^
No SVP	15.91 (.010)	1.00 REF	21.36 (.005)	1.00 REF	
SVV **	44.45 (.029)	3.09 (2.63–3.64)	64.11 (.016)	7.10 (6.42–7.87)	17.23 ^P < .0001^
No SVV	14.36 (.008)	1.00 REF	9.02 (.005)	1.00 REF	

*djusting for Sexual violence victimization (SVV); ** Adjusting for Sexual violence perpetration SVP)

aPRR = Adjusted prevalence rate ratios adjusting for current relationship status (yes v no), race (minority v majority), condition (intervention v control), sexual attraction (exclusively attracted to opposite sex: yes vs no: sexual minority), witnessed parental partner violence (yes v no)

95% CI = (95% confidence interval around the aPRR); REF = referent group; *SE* = standard error. NOTE: aPRR analyses provide comparisons with the referent group being no SVP nor SVV. Test of Interaction Sex * SV measure (Score Statistics For Type 3 GEE Analysis). p-values < .001 were determined to be statistically significant given the large sample size

## Results

First, we investigated sex differences in rates of any form of SVV and SVP overall and by SV tactic (Table 1). Consistent with prior research, rates of any SVP among males (10.8%) were twice that of females (5.2%), while rates of any SVV were higher among females (21.2%) than males (13.3%). For both sexes, the most common type of SVV tactic experienced was coerced sex (males: 8.4%, females: 15.8%) followed by alcohol/drug facilitated or incapacitated sex (males: 8.2%, females: 9.3%), and physically forced sex (males: 4.7%, females: 5.5%). With regard to SVP tactics, both males and females reported using alcohol/drug facilitated or incapacitated sex at the highest rate (males: 8.5%, females: 4.1%), followed by sexual coercion (males: 5.3%, females: 2.0%), and physically forced sex (males: 4.3%, females: 1.4%).

We next examined demographic or violence risk factors that were associated with SVV and/or SVP. For both sexes, correlates of increased SVV and SVP rates included sexual minority status (not exclusively attracted to the opposite sex), being in a sexual or romantic relationship, and witnessing parental IPV. Non-White race was an additional correlate of increased SVP for both sexes and of SVV for males only. Subsequent analyses investigating SV and binge drinking (Table 2) and problem alcohol use (Table 3) were adjusted for race, sexual attraction, relationship status, and witnessing parental IPV.

For the primary analyses, we investigated sex differences in the association between SV and current binge drinking and alcohol problems overall and by tactic. Given observed sex differences in the effect of SVV and SVP on both binge drinking and alcohol problems, all analyses were presented by sex where interaction terms in multivariable logistic regression models were used. Males were more likely (*p* < .001) to disclose binge drinking in the past month (26.4% in males and 24.1% in females; Table 2) yet no sex differences in alcohol problems were noted (12.8% in males and 12.7% in females; see Table 3).

Hypothesis 1 stated that adolescents (both male and female) experiencing SVV or using SVP would report more frequent binge drinking and alcohol problems compared to adolescents reporting no SV. Consistent with this hypothesis, those who experienced SVV or used SVP were more likely to disclose binge drinking in the past month and alcohol problems in the past 12 months (Tables 2 and 3). This pattern held for adolescent males and females, across increasing numbers of SV tactics, and within each of the three specific SV tactics. For both male and female adolescents, the magnitude of associations between SV (SVP and SVV) and alcohol problems (adjusted PRRs) were larger than associations between SV and binge drinking. Hypothesis 2 stated that SVP was expected to be more strongly associated with binge drinking and alcohol problems among males compared to females. Results were partially consistent with this hypothesis. As displayed in Table 3, SVP was more strongly associated with alcohol problems among males (female aPRR = 1.83; male aPRR = 2.63; test for interaction: X^2^ = 10.57, *p* = .001), but there were no sex differences in the association between SVP and binge drinking. Although not specifically hypothesized, sex differences were also found in the associations between SVV and both alcohol outcomes. Binge drinking was more strongly associated with SVV among females (aPRR = 1.96) compared to males (aPRR = 1.40; test for sex x SV interaction; X^2^ = 12.61, *p* < .0001). This pattern held for number of SV tactics (p < .0001) and all three SVV tactics. A similar sex difference pattern was observed for alcohol problems, where any SVV was more strongly associated with alcohol problems among females (female aPRR = 4.50; male aPRR = 2.70; test for interaction: X^2^ = 11.91, p < .0001).

Our examination of specific SVV and SVP tactics proved to be important (see Table 3). Hypothesis 3 stated that for both male and female adolescents, drug/alcohol-facilitated or incapacitated SV (SVP and SVV) was expected to be the tactic most strongly linked to binge drinking and alcohol problems. Although we anticipated that drug/alcohol-facilitated or incapacitated SV would be most strongly linked to both SVP and SVV among males and females, there was an important interaction between SV (SVP vs SVV) and sex (male vs female). With regard to SVP, we observed higher adjusted prevalence rate ratios (PRR) for males with regard to both current binge drinking (male aPRR = 1.82, female aPRR = 1.29; X^2^ = 17.42 p < .0001) and alcohol problems (male aPRR = 2.52, female aPRR = 1.27; X^2^ = 15.22, p < .0001) associated with drug/alcohol-facilitated or incapacitated SVP. In contrast, drug/alcohol-facilitated or incapacitated SVV was associated with significantly higher rates of binge drinking (female aPRR = 2.81, male aPRR = 1.66; X^2^ = 14.22, p < .0001) and alcohol problems (female aPRR = 7.10, male aPRR = 3.09; X^2^ = 17.23, p < .0001) in females compared to males. There were no significant sex differences in the relation between coerced or forced SVP or SVV and either alcohol outcome (all ps > .001).

## Discussion

The present study was the first population-based study to examine sex differences in the effects of past 12-month SVV and SVP (overall and by SV tactic) on current binge drinking and alcohol problems in male and female high school students. As expected, both SVV and SVP were associated with an increased frequency of binge drinking and alcohol problems among males and females. This was true across increasing numbers of SV tactics and within each SV tactic. Of note, for both male and female adolescents, SVV and SVP were more strongly linked to alcohol problems than to current binge drinking. This is consistent with a robust literature demonstrating that alcohol use behavior (i.e., frequency/quantity of use) and alcohol-related problems (e.g., social/academic/occupational impairment, physiological dependence) are predicted by different risk factors, display different trajectories, and demonstrate distinct relationships with other relevant constructs (Cooper et al. 1995; Read et al. 2003) including victimization history and trauma-related mental health problems (Read et al. 2013; Goldstein et al. 2010). A prospective study is needed to determine the time-course of associations between SV and both binge drinking and alcohol problems. There is some evidence that past alcohol use behavior (including binge drinking) is the most robust predictor of future binge drinking, regardless of SV; though most of this work has been conducted in relation to SVV only (McCauley et al. 2009; Testa and Livingston 2000; Testa et al. 2007). In contrast, preliminary evidence suggests that new SVV is linked to subsequent alcohol problems above and beyond prior SVV and alcohol problem history (Kilpatrick et al. 1997), suggesting that perhaps alcohol problems are more robustly linked to SV than alcohol consumption. SV and alcohol-related problems may be bi-directionally related, and potentially mediated by acute increases in alcohol consumption (Cooper et al. 1995; Read et al. 2013).

Sex differences in the effects of SVP on binge drinking and alcohol problems were posited and observed specifically with regard to drug/alcohol-facilitated or incapacitated sex. When considering this form of SV, the association between SVP, adjusting for SVV, and both alcohol-related measures was stronger among males compared to females, while SVV, adjusting for SVP, was more strongly associated with both alcohol outcomes among females relative to males. With regard to SVP, these findings are in line with a model wherein male adolescents who engage in frequent binge drinking may be at increased risk for SVP, particularly if a potential sexual partner is also drinking to intoxication. In such contexts, potential perpetrators may misinterpret sexual cues (e.g., perceive female alcohol use as an indicator of sexual interest; Abbey et al. 2000) or attend less to cues that inhibit sexually aggressive behavior in favor of more immediately salient feelings of sexual arousal, frustration, or entitlement (Abbey 2011; Gallagher et al. 2010). Among male college students, there is some evidence that intentional use of alcohol or drugs to incapacitate a victim is associated with higher typical levels of alcohol consumption, more favorable attitudes toward casual sex, and consumption of a higher volume of alcohol/increased intoxication level proximal to committing SVP (Abbey 2011). To better evaluate this model among adolescents, future studies should consider typical drinking behavior and alcohol problems prior to engaging in SVP, general attitudes regarding sexual partners and expectancies surrounding alcohol use, and the impact of proximal alcohol use and intoxication on SVP by tactic. Separating predictors of intentional drug/alcohol facilitated SVP from incapacitated SVP are encouraged given the involuntary nature of substance use in the former.

Although SVV was more strongly associated with binge drinking and alcohol problems among females in the present study, sex differences in these relations were not specifically hypothesized a priori. This was because prior findings have been mixed regarding whether such sex differences actually exist (Dube et al. 2005; Fergusson et al. 2013; Garnefski and Arends 1998; Howard and Wang 2005; Loh et al. 2014). Methodological differences may account for some of the discrepancies across studies, as investigations have differed in their operationalization of SVV (e.g., childhood sexual abuse versus recent adolescent SVV), and few studies have considered the role of SV tactics or prior levels of binge drinking/alcohol problems. Conflicting findings may also be due, in part, to unmeasured mediators or moderators of the relation between SVV and alcohol-related outcomes that differ by sex. For example, following SVV, both adolescent and adult females are more likely than males to develop posttraumatic stress disorder (PTSD), even when considering the disproportionate risk for experiencing SVV borne by females (see Kilpatrick et al. 2017 for a review). Moreover, among young adult college students, different mechanisms may link PTSD symptoms to alcohol-related outcomes in males (e.g., difficulty with impulse control, delaying gratification) versus females (e.g., difficulty engaging in goal-directed behavior when distressed) though these findings need to be extended to an adolescent sample (Boyraz et al. 2017; Tripp et al. 2015).

Of note is our finding sex differences were specific to drug/alcohol-facilitated or incapacitated sex, yet no sex differences in the impact of SVV or SVP on either alcohol-related outcome for sexual coercion or physically forced sex. This is in line with previous findings documenting unique risk factors and associated sequalae of drug/alcohol-facilitated or incapacitated sex compared to other SV tactics among adults. With regard to SVV among women, Testa et al. (2003) found alcohol use prior to age 18 to be a unique risk factor for incapacitated SVV, and drug/alcohol-facilitated or incapacitated SVV was more likely to occur proximal to when a woman spent time at a bar or party. Several studies have also found that women with a history of drug/alcohol-facilitated or incapacitated SVV report higher levels of alcohol use (including binge drinking) and alcohol problems (Abbey et al. 2004a, b; Kaysen et al. 2006; McCauley et al. 2009; Testa et al. 2003). Indeed, McCauley et al. (2009) found that college women with a history of drug/alcohol-facilitated or incapacitated SVV, but not forcible SVV, had an increased odds of past-year binge drinking and other substance abuse. With regard to SVP among men, work by Kingree and Thompson (2015) among a prospective cohort of 638 first year male college students examined several risk factors for SVP and found that only binge drinking prospectively predicted alcohol-facilitated SVP, while impulsivity, rape myth attitudes, and hostility toward women predicted non-alcohol-involved SVP. To our knowledge, our study is the first to document sex differences in the strength of the relationship between SV and alcohol-related outcomes, and to note that such sex differences are unique to drug/alcohol-facilitated or incapacitated SV tactics. Additional research is needed to better understand the role of alcohol and other substance use prior to, during, and after experiences of SVV and SVP.

A specific strength of this study was its ability to examine the impact of combined experiences of SVV/SVP. Rates of binge drinking, and especially alcohol problems, were quite high among males and females who reported both SVV and SVP. Of note, sex differences in the prevalence of the alcohol outcomes as a function of any SV were no longer significant when considering SVV and SVP combinations. As the majority of extant research on risk factors associated with SV have focused on SVV in females and SVP in males, we know very little of the unique characteristics of individuals who have both experienced SVV and engaged in SVP. This may be a population that is uniquely at risk for alcohol problems as well as other forms of psychopathology, and research is needed to better understand the nature of SV among this group.

The cross-sectional nature of the present data precludes temporal inferences regarding associations between SV and alcohol-related outcomes. Binge drinking and alcohol problems may temporally precede, and serve as a distal risk factor for SV. For example, higher general binge drinking or alcohol problems may increase risk for SVV by more frequently exposing youth to high risk situations where SVV is likely to occur (Gidycz et al. 2007; Mouilso et al. 2012). Typical alcohol use may also increase risk for SVP, as heavy drinking frequently occurs in social situations that are likely to lead to SVP (e.g., parties, bars), heavy drinkers may use intoxication as a means to justify inappropriate or sexually aggressive behavior, and certain personality traits such as impulsivity may increase risk both for drinking and SVP behavior (Abbey et al. 2001). Alcohol intoxication may also serve as a proximal risk factor for SV by leading to reduced consent and refusal capacity and decreased risk recognition in the case of SVV (Davis et al. 2009; Testa et al. 2000) and by resulting in impaired executive functioning and response inhibition as well as misperception of sexual interest in the case of SVP (Abbey 2011; Abbey et al. 2000; Gallagher et al. 2010). Alternatively, SV may increase risk for binge drinking or alcohol problems. This risk pathway has been primarily examined in relation to women experiencing SVV, and increased drinking (including binge and heavy drinking) may serve as a method of coping with distress associated with prior SVV experiences (Kaysen et al. 2006; Norris et al. 2019; Parks et al. 2014). In short, binge drinking and alcohol problems may be a consequence of and risk factor for SVV and SVP, that may differentially impact young men and women. Evaluation of sex differences in the complex relationship between SV and alcohol-related outcomes is challenging given that large samples are needed to obtain adequate numbers of males with a history of SVV and females with a history of SVP. Further these samples must be followed prospectively to establish correct temporal sequencing. An adequately powered longitudinal study of adolescents examining sex differences in prospective associations between SV and binge drinking/alcohol problems by SV tactic is needed. Additional limitations included our exclusive reliance on self-reports of SV and alcohol use/problems. This information may be limited by recall of experiences, current distress, or unwillingness to disclose experiences of SV or alcohol use/problems. Moreover, only students in public high schools in one state were included, thus despite the large sample size, the findings may not generalize to different samples.

With these challenges fully acknowledged, the contribution of the current paper includes our examination of sex differences in the association of SVV and SVP on both current binge drinking and alcohol problems. The majority of studies investigating correlations with SVV and binge drinking (or alcohol problems) have been conducted among young college-aged women and those focused on SVP have been largely limited to college-aged men. Our ability to investigate SVV and SVP within the three SV tactics is also a contribution. Our findings suggest differing SV effects on both binge drinking and alcohol problems by SVV/SVP tactic with alcohol/drug facilitated or incapacitated sex having a much stronger and unique association for males versus females with both binge drinking and alcohol problems than that for sexual coercion and physically forced sex.

In sum, both SVV and SVP were associated with increased rates of current binge drinking and alcohol problems among a large sample of male and female adolescents. Interventions to reduce rates of SV, such as those in the parent randomized control trial on which this analysis is based (Coker et al. 2017), may result in reduced alcohol use or problems by reducing SV. Additionally *Stop SV: A Technical Package to Prevent Sexual Violence* developed by the CDC provides evidence-based programs, policies and practices to address the perpetration and victimization behaviors identified in this study (Basile et al. 2016). Finally, the recent toolkit (Klein et al. 2018) addressing alcohol’s role in sexual violence in college settings is an excellent resource to address these frequently co-occurring health threats for adolescents and young adults.
